# An effective fragment-based dual conditional diffusion framework for molecular generation

**DOI:** 10.1093/bib/bbaf727

**Published:** 2026-01-19

**Authors:** Haotian Chen, Yiting Shen, Jichun Li, Weizhong Zhao

**Affiliations:** Hubei Provincial Key Laboratory of Artificial Intelligence and Smart Learning, Central China Normal University, Wuhan, Hubei 430079, PR China; School of Computer, Central China Normal University, Wuhan, Hubei 430079, PR China; National Language Resources Monitoring & Research Center for Network Media, Central China Normal University, Wuhan, Hubei 430079, PR China; Detroit Green Technology Institute, Hubei University of Technology, Wuhan, Hubei 430079, PR China; School of Computing, Newcastle University, Newcastle upon Tyne NE4 5TG, United Kingdom; Hubei Provincial Key Laboratory of Artificial Intelligence and Smart Learning, Central China Normal University, Wuhan, Hubei 430079, PR China; School of Computer, Central China Normal University, Wuhan, Hubei 430079, PR China; National Language Resources Monitoring & Research Center for Network Media, Central China Normal University, Wuhan, Hubei 430079, PR China

**Keywords:** fragment-based molecular generation, structure-based drug design, conditional diffusion model

## Abstract

Fragment-based molecular generation has emerged as a promising paradigm in structure-based drug design (SBDD), deriving effective compounds with advanced properties, including chemical validity, synthetic feasibility, pharmacological relevance, etc. However, existing approaches often struggle with generating molecules which can both conform to 3D structural constraints and retain chemical plausibility. This is largely due to the fact that prior works often treat scaffolds and R-groups of molecules indiscriminately, overlooking the distinct semantic roles played by scaffolds and R-groups. Specifically, the scaffold serves as the rigid structural backbone that determines the global geometric topology and binding pose, whereas R-groups act as functional substituents responsible for fine-tuning local physicochemical interactions. Therefore, in this work, we propose fragment-based dual conditional diffusion (FDC-Diff), a novel dual conditional diffusion framework that integrates chemical priors and structural cues for fragment-based molecular generation. Unlike traditional *de novo* methods that generate atoms sequentially, FDC-Diff decomposes the molecule generation process into two semantically complementary stages. Given the protein pocket and an initial fragment, in the first stage, a spatially constrained scaffold is constructed to capture the global molecular topology. In the second stage, R-groups onto the obtained scaffold are elaborated to capture local semantics to further refine molecular properties. To ensure synthetic accessibility, initial fragments and scaffold-modification hierarchy are derived from curated reaction rules, and a physical-chemistry-inspired refinement step is applied to optimize final conformations. Experimental results on multiple SBDD benchmarks demonstrate that FDC-Diff achieves state-of-the-art performance in terms of comprehensive evaluations. Furthermore, our model excels at producing chemically valid, spatially compatible, and pharmacologically relevant molecules, suggesting its potential as a feasible tool for fragment-based drug design.

## Introduction

Recent years have witnessed the rapid development of artificial intelligence-assisted drug design (AIDD) technology, which provides powerful tools for discovery and optimization of small molecule drugs [1, 2]. Within the generative strategy-based models of AIDD, *de novo* molecular design [3–7] has become a popular paradigm. Based on the representation granularity for compounds, *de novo* molecular design methods can be roughly classified into two families: atom-based and fragment-based [7]. Generally, atom-based *de novo* design generates molecules directly at the level of atoms and bonds, incrementally assembling structures while enforcing chemical constraints (e.g. valence, ring closure, and stereochemistry) and determining connectivity on the fly. In contrast, fragment-based *de novo* design constructs molecules by linking or extending chemically meaningful fragments, which typically starts from known or validated active fragments and generates remaining components reasonably by integrating knowledge from structural biology and computational analysis [8–11]. Since fragment-based methods are more aligned with the actual drug development process [8–11], this study focuses on the molecular generation from this line of *de novo* molecular design.

**Figure 1:**
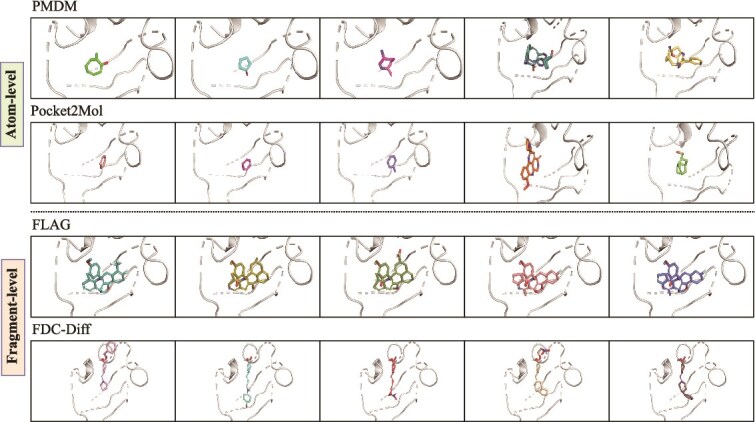
Visualization of molecules generated by different methods. (i) Atom-level methods (PMDM, Pocket2Mol): These methods generally exhibit a tendency to generate molecules with a low number of heavy atoms and simple structures, resulting in generated molecules lacking sufficient pharmacophoric features to maintain biological activity. Furthermore, in an attempt to fill larger protein pocket cavities, the Pocket2Mol model tends to generate large polycyclic systems, which lack physical realism. (ii) Fragment-level methods: FLAG tends to stack large, rigid fragments, facing significant challenges in geometric refinement. Errors during each fragment generation step accumulate, ultimately leading to the collapse of the molecular structure. FDC-Diff generates drug-like molecules with appropriate heavy atom counts. By ensuring coherent global topology alongside precise local geometry, it significantly mitigates the risk of generating physically invalid structures.

In recent years, researchers have proposed several fragment-based drug design methods based on different strategies [2, 12–15]. However, existing fragment-based methods [16–18] have the following limitations: (i) 2D representations fail to capture 3D conformations and pocket context, posing challenges for direct structure-based design [18, 19]; (i) most methods adopt bottom-up assembly (i.e. constructing molecules fragment by fragment), which is prone to error accumulation, showing limited global control over functional regions and synthetic accessibility (SA). This may ultimately lead to the collapse of the molecular structure, preventing the formation of a stable and reasonable 3D conformation [19, 20]. This issue can be illustrated by examples shown in Fig. 1.

To address these structural failures, we argue that generative models must align with the intrinsic hierarchical nature of medicinal chemistry. From a structure–function perspective, a drug-like molecule is not a uniform collection of atoms but a hierarchical entity consisting of two distinct components. A molecule typically contains a relatively stable core region that determines the overall topology, provides a connective framework to integrate various functional groups or fragments, and governs target-binding characteristics, as well as a peripheral modification region responsible for regulating the physicochemical and pharmacological properties of the molecule [21, 22]. These two components differ significantly in structural complexity, functional roles, and synthetic strategies, as well as in the ways computational models handle them. The biases and error-prone assembly in existing methods often stem from treating core and peripheral elements uniformly, leading to suboptimal exploration of novel fragments (especially in peripheral regions) and insufficient global stability enforced by the core. For example, as shown in Fig. 1, due to the lack of essential chemical prior knowledge, some models tend to generate invalid structures. Effectively distinguishing between these core and peripheral regions within molecular structures is crucial for further improving the precision and controllability of molecular generation processes [23–27].

**Figure 2:**
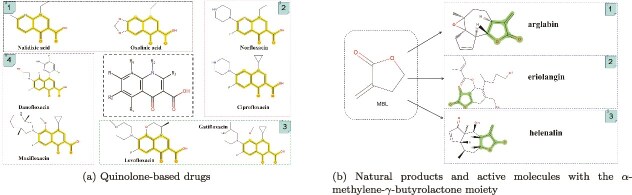
Illustration of distinct roles of scaffold and R-groups in molecular design. (a) Quinolone-based drugs with the bicyclic heterocycle core structure. The substituents at position 3 (COOH) and position 4 (=O) are critical for the drug’s binding to enzymes, making them common features of Quinolone-based drugs. The remaining substituents are related to the drug’s antimicrobial properties, side effects, and metabolic characteristics. (b) Natural products and active molecules containing the $\alpha $-methylene-$\gamma $-butyrolactone moiety. The $\alpha $-methylene-$\gamma $-butyrolactone moiety can form covalent bonds with biomacromolecules through 1,4-conjugate addition, thereby influencing the biological function of these molecules. This moiety is regarded as a pharmacophore with a covalent binding mode.

To illustrate this distinction, Fig. 2a shows the evolution of the quinolone lineage, for which the bicyclic heterocycle works as the core structure. More specifically, early non-fluorinated quinolones (e.g. nalidixic acid) were primarily used to treat Gram-negative urinary tract infections due to their limited spectrum and poor pharmacokinetics. With the introduction of a C-6 fluorine atom and a C-7 piperazinyl substituent, significant improvements were obtained in both the drug’s antibacterial activity and pharmacokinetic properties, resulting in the development of more effective agents (e.g. norfloxacin and ciprofloxacin). Later respiratory quinolones (e.g. levofloxacin and gatifloxacin) and their analogs (e.g. moxifloxacin and gemifloxacin) further extended the coverage to Gram-positive bacteria and anaerobes, while also improving the drug safety profiles [28–30]. In this context, the bicyclic heterocycle scaffold is essential for defining the drug class, establishing the fundamental mechanism of action, and providing the molecular framework for antibacterial activity. In contrast to the scaffold’s structural and functional role, Fig. 2b presents the $\alpha $-methylene-$\gamma $-butyrolactone (MBL) pharmacophore and its examples from various natural products, emphasizing the structural characteristics of MBL as a substituent. As a Michael acceptor, MBL forms covalent bonds with nucleophilic residues, which modulate the activity of specific biological targets. MBL is commonly found in bioactive natural products such as helenalin, arglabin, and eriolangin [31]. Within this framework, the R-group is pivotal in determining the drug’s potency, selectivity, and covalent binding ability. Generally speaking, examples in Fig. 2 highlight the distinct roles of the scaffold and R-group in molecular design, i.e. the scaffold preserves the structural integrity while the R-group enhances the drug’s properties, such as efficacy, targeting ability, and safety.

Based on the observations mentioned above, in this study, we propose a novel fragment-based dual conditional diffusion model, termed FDC-Diff, for structure-based drug design (SBDD). This framework decomposes the molecular generation process into two synergistic substages based on preconstructed molecular fragments, which are responsible for capturing the global and local semantic information of molecules, respectively. First, we preprocess existing small molecules to obtain chemically meaningful fragments generated based on validated reaction rules and decompose existing bioactive molecules based on specific chemical reaction templates. Then, in the first generation stage, FDC-Diff focuses on constructing a 3D structure that satisfies the spatial constraints of the protein binding pocket. This structure undertakes the global topology and spatial anchoring of the molecule, which will determine the overall shape, binding mode, and potential growth sites of key pharmacophoric groups. Next, in the second generation stage, FDC-Diff learns to continue molecular growth on the obtained scaffold by refining local chemical environments. This stage is designed to optimize specific local properties (e.g. hydrophilicity and solubility) enabling functional modification and fine-tuning of physicochemical attributes. Distinct from holistic generation approaches, this stepwise progression allows FDC-Diff to balance macro-level geometric constraints with micro-level chemical validity. While previous methods often struggle to satisfy both criteria simultaneously, our framework’s decoupled nature allows for targeted optimization at each scale. Below, we discuss the specific advantages of this architecture compared with existing methods.

The key distinction from previous methods lies in FDC-Diff’s innovative integration of fragment-based preprocessing with a dual-stage conditional diffusion paradigm and a graph neural network (GNN)-based bond reconstruction mechanism. Specifically, unlike Pocket2Mol [32], which relies on atom-by-atom autoregressive sampling that can suffer from inefficiency and invalid structures due to sequential dependencies, FDC-Diff leverages cross-stage conditional diffusion to generate fragments in a more parallelized manner, enhancing computational efficiency, molecular validity, and adherence to protein pocket constraints. In contrast to PMDM [33], an atom-level diffusion model that starts from random noise and may struggle with capturing chemically meaningful intermediates, FDC-Diff initiates diffusion at the fragment level using preconstructed, reaction-rule-validated fragments, then incorporates global scaffold construction for spatial anchoring followed by local refinement for physicochemical optimization and GNN-driven bond assembly to ensure precise, stable molecular topologies. Compared with FLAG [18], a fragment-based autoregressive method, which generates the molecule in a single sequential stage by retrieving fragments from a fragment library for assembly, FDC-Diff decomposes the process into synergistic global and local stages, enabling improved control over the generation stage and reducing the risk of generating meaningless structures.

In conclusion, our approach offers the following key contributions:

- **Semantic Disentanglement of Geometry and Properties** FDC-Diff strategically decouples the generation process into two cooperative subtasks. We prioritize the scaffold to satisfy global geometric constraints (spatial anchoring) and subsequently elaborate R-groups to tune local physicochemical properties. This hierarchical distinctness ensures that generated molecules possess both precise 3D pocket adaptability and reasonable chemical distributions.
- **Reaction-Informed Dataset and FBDD-Guided Initialization** To bridge the gap between generative AI and practical synthesis, we construct the scaffold-R-group dataset via reaction-based rules and employ BRICS for chemically valid fragmentation. Crucially, we utilize fragments adhering to the Three Principles of Fragment-Based Drug Design (FBDD) as the specific starting points for generation. By embedding these chemical priors directly into the process, FDC-Diff incorporates a strong inductive bias, ensuring that both the building blocks and the resulting compounds adhere to synthetic validity and structural integrity.
- **State-of-the-Art Performance and Practical Utility** Extensive empirical evaluations across multiple benchmark tasks demonstrate that FDC-Diff significantly surpasses existing baseline methods. FDC-Diff achieves superior results on essential evaluation metrics, including binding affinity, quantitative estimation of drug-likeness (QED), and SA. These findings not only verify the effectiveness of the proposed framework but also highlight its substantial potential for practical deployment in fragment-based drug discovery.

## Related work

In this section, we briefly review related work to this study, including several representative methods of atom-based and fragment-based *de novo* molecular design methods.

### Atom-based molecular design

For atom-based *de novo* molecular design, molecules are typically represented as SMILES strings or molecular graphs, and then generated or constructed by iteratively adding atoms and bonds [7, 15, 32–37]. For example, Peng *et al.* [32] proposed Pocket2Mol, in which a pocket-conditioned, $E(3)$-equivariant autoregressive scheme is employed for molecular generation. Specifically, Pocket2Mol predicts the new atom’s relative coordinates using a Gaussian mixture model and infers element type and bond order through equivariant attention. Pocket2Mol effectively couples 3D geometry with bonding information, thereby reducing the accumulation of long-range errors. Following the same autoregressive paradigm for molecular generation, GraphBP employs a 3D GNN to encode both the context of the pocket and placed atoms. It uses a local reference atom to define a spherical coordinate system and employs a flow-based head for sequential prediction of the next atom’s type and $(r,\theta ,\phi )$ coordinates. This design inherently ensures $\mathrm{SE}(3)$ equivariance while guaranteeing chemically valid atom placement. In addition, PMDM [33] adopts a pocket-conditioned dual-diffusion formulation with $\mathrm{SE}(3)$-equivariant dynamics. By combining local and global encoders with cross-attention mechanisms, PMDM effectively captures both the semantic and spatial context of proteins, enabling the generation of highly potential and drug-like molecules.

However, there are some drawbacks in existing atom-based molecular design methods: (i). Some approaches [16, 34, 37] still rely on 2D graphs or treat molecules independently of protein pockets, limiting their ability to design receptor-specific, high-affinity molecules; (ii). Some methods [32, 33] fail to effectively integrate chemical prior knowledge, leading to unstable generative quality. These limitations typically manifest in two distinct ways: first, producing molecules with fewer heavy atoms, which results in overly simplistic structures that lack pharmacological value; second, generating chemically invalid topologies (e.g. distorted fused ring systems and unrealistic geometries), which might lead to physically unreasonable structures. These issues are illustrated in Fig. 1.

### Fragment-based molecular design

Fragment-based molecular design treats chemically meaningful fragments as building blocks and assembles molecules by linking or growing fragments under explicit rules, thereby improving chemical validity [15]. Following the coarse-to-fine paradigm, the Junction Tree VAE (JT-VAE) [38] ensures graph-level validity through a two-stage generative process. First, it creates a tree-structured scaffold composed of valid substructures, such as rings and bonds. Then, it assembles these substructures into a complete molecular graph by using a message-passing mechanism. This coarse-to-fine generation process ensures the chemical validity of the molecules at every step, and the model efficiently handles large molecular graphs by leveraging substructures. Unlike the treatment of JT-VAE, DeepFrag [16] takes the fragment-based lead optimization as a discriminative fragment-completion task. Specifically, DeepFrag first analyzes the 3D voxelized representation of the receptor–ligand complex, and then utilizes a deep convolutional neural network to predict the molecular fragment that should be added after removing a fragment, thereby completing the ligand structure. This approach allows for rapid and efficient identification of suitable fragments, which can be used to improve the binding affinity of the ligand to the target receptor. Zhang *et al.* [18] proposed FLAG, in which fragment priors are introduced into SBDD and ligands are generated fragment by fragment directly in 3D under pocket conditioning. The advantages of FLAG lie in that it can not only ensure bond lengths and angles conform to cheminformatics rules, but also can improve both efficiency and accuracy.

Despite the success of fragment-based generation methods in certain applications, they still have the following limitations. First, some methods [38] are designed on 2D representations and overlook 3D conformations and pocket context, which complicate direct structure-based design [18]. To address this limitation, FDC-Diff introduces a fragment-driven diffusion framework that models the 3D structure based on Cartesian coordinates. This approach allows for better consideration of 3D conformations and target context, overcoming the limitations of traditional 2D representations. Second, some methods [18] employ autoregressive generation techniques without considering the prior constraints of the scaffold structure, which can lead to a more random and less controllable process, generating unreasonable structures (e.g. polycyclic molecules, as shown in Fig. 1), resulting in chemical instability and irrationality [19, 20]. In contrast, FDC-Diff employs a conditional diffusion model to separately model the global scaffold structure and local functional group information, enhancing the controllability of the generation process. It ensures the stability and rationality of the global structure, thereby reducing the risk of generating unreasonable structures.

## Preliminaries

In order to describe the proposed framework conveniently, in this section, we provide a brief introduction to diffusion model and the definition of the conditional molecular generation.

### Brief introduction to diffusion model

The diffusion model [39] consists of two Markov chains: the forward (diffusion) process and the reverse (denoising) process. The diffusion process progressively adds Gaussian noise to the data following a variance-preserving schedule, while the reverse process refines the noisy data, eventually recovering the original data by removing the noise. The key goal of diffusion model is to learn the reverse process by using a parameterized neural network.

The forward diffusion step can be formulated as


(1) \begin{align*}& q(x_{t} \mid x_{t-1}) = \mathcal{N}\!\left(x_{t};\, \sqrt{1-\beta_{t}}\,x_{t-1},\, \beta_{t} I\right)\end{align*}

where $x_{t}$ denotes the noisy representation at time step $t$, $\beta _{t}$ is the noise variance determined by the schedule, and $I$ is the identity matrix. The noise variance typically follows a linear schedule, with $\beta _{t}$ increasing from $10^{-4}$ to $0.02$ across a total of $T$ diffusion steps.

The reverse process is parameterized by a neural network that estimates the noise:


(2) \begin{align*}& p_\theta(x_{t-1}\mid x_{t}) = \mathcal{N}\!\left(x_{t-1};\, \mu_\theta(x_{t},t),\, \Sigma_\theta(x_{t},t)\right)\end{align*}

where $\mu _\theta (x_{t},t)$ and $\Sigma _\theta (x_{t},t)$ denote the mean and covariance predicted by the model, respectively. The objective of training procedure is to minimize the following variational bound:


(3) \begin{align*}& L = \mathbb{E}_{x_{t}, x_{0}, \epsilon}\Big[ \, \| \epsilon - \epsilon_\theta(x_{t}, t) \|^{2} \,\Big]\end{align*}

where $\epsilon $ is the injected Gaussian noise and $\epsilon _\theta (x_{t},t)$ is the noise predicted by the parameterized model.

### Definition of conditional molecular generation

Conditional molecular generation is a task to design molecules with desired structures and functions based on specific conditions or constraints. It leverages input conditions, such as physicochemical properties, target binding affinity, or toxicity of molecules, to guide the generation process. By effectively controlling this process, conditional molecular generation is able to design molecules that meet specific functional requirements.

In this study, we extend the existing framework by incorporating a conditional diffusion to handle the molecular generation process. The diffusion model is formulated as a gradual denoising process, where external conditions guide the generation of molecules at each step [35]. Specifically, in the task of 3D molecular generation, diffusion models must handle both discrete atom types and continuous atomic coordinates. Molecules are represented as $ G^{L} = (\mathbf{x}, \mathbf{r}) $, where $ \mathbf{x} \in \{0,1\}^{n \times f} $ denotes the one-hot encoded matrix of atom types for $ n $ atoms over $ f $ distinct types, and $ \mathbf{r} \in \mathbb{R}^{n \times 3} $ represents the corresponding Cartesian coordinates of the atoms. Additionally, we incorporate the information about the protein pocket which is denoted as $ G^{P} = (\mathbf{x}^{P}, \mathbf{r}^{P}) $, as well as molecular fragments $ F = (\mathbf{x}^{F}, \mathbf{r}^{F}) $ with anchor atoms $ \mathcal{A} \subseteq \{1, \dots , n_{F}\} $ that identify potential growth sites for the fragment. The scaffold information is represented as $ G^{S} = (\mathbf{x}^{S}, \mathbf{r}^{S}) $ and the R-group information is represented as $ G^{R} = (\mathbf{x}^{R}, \mathbf{r}^{R}) $. The conditional information is represented as $G^{C}$, which in the scaffold stage is denoted as $ G^{C}_{S} = (G^{P}, F, \mathcal{A}) $, and in the R-group stage is denoted as $ G^{C}_{R} = (G^{P}, G^{S})$. The goal is to learn the conditional distribution:


(4) \begin{align*}& p_{\theta}(G^{L}_{t-1} \mid G^{L}_{t}, G^{C})\end{align*}

**Figure 3:**
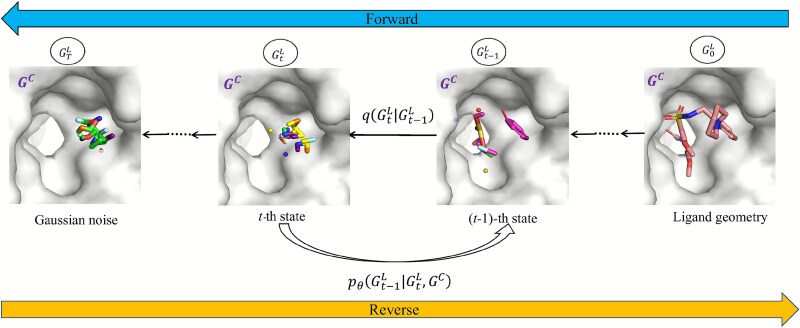
Illustration of the conditional generation process. The forward process gradually adds noise to the molecular structure, while the reverse process denoises it to generate chemically valid molecules.

By doing so, we generate molecules that not only satisfy specific functional and structural constraints but also allow for optimization of molecular properties based on the given conditions. An overview of the conditional diffusion process is presented in Fig. 3.

## Method

**Figure 4:**
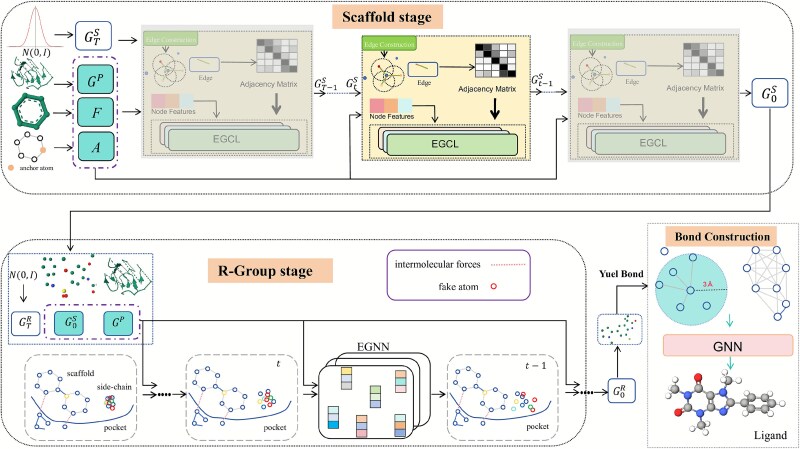
Overview of the proposed dual conditional generation framework. The scaffold stage generates a molecular skeleton from a fragment and anchor atoms, and the R-group stage completes the molecule based on the derived scaffold. Finally, the generated atomic coordinates and atom types are input into the YuelBond framework for bond construction, deriving a complete molecule.

In this section, we elaborate on the proposed molecular generation framework. The overall architecture is shown in Fig. 4. The framework adopts a dual conditional diffusion model, and each stage models different substructures in the 3D molecular structure generation process. In the first stage, the initial skeleton conformation is generated based on the input molecular fragment, growth sites, and protein pockets. In the second stage, the spatial structure of the skeleton is further expanded and refined to generate the complete molecular conformation. Finally, the generated atomic coordinates and atom types are fed into a bond construction tool to ultimately generate a complete 3D molecular structure that closely matches the target protein pocket.

### Scaffold generation stage

The molecular scaffold typically constitutes the structural skeleton of a compound, determining its overall geometric layout and fundamental chemical functionality. In the task of molecular generation, the rationality of the scaffold directly impacts the extensibility of the structure as well as the pharmacological relevance and SA of the resulting molecule. Therefore, constructing a reasonable scaffold serves as the critical first stage in the molecular generation pipeline.

For the scaffold generation stage, it requires careful consideration of several key aspects: (i) generating a spatially continuous and chemically reasonable distribution of atomic coordinates to form a well-defined structural framework; (ii) maintaining spatial regularities, such as plausible bond lengths and bond angles, to facilitate subsequent structural reconstruction and refinement; and (iii) incorporating geometric constraints from the protein pocket during the generation process to ensure spatial alignment and functional compatibility with the target binding sites. To systematically achieve the effective scaffold construction and functional guidance, we decompose this stage into multiple steps, as detailed below.

#### Forward process

In the diffusion model for molecular generation, the forward noise injection process aims to gradually transform the initial structure into a noisy state. Initially, the atomic coordinates of the fragment $ F $ remain fixed. Gaussian noise is then applied to the atoms designated for expansion, denoted as $G^{S}_{T}$, perturbing their spatial positions and types. As the noise is progressively added, the relative positions and types of the atoms change, gradually blurring the originally defined molecular structure and ultimately forming a starting structure with appropriate randomness. In order to present the following formal descriptions conveniently, we use the symbol $\bar{F}$ to replace $G^{S}_{T}$ in the following subsection.

#### Denoising process

In the denoising process, the anchor atoms $ \mathcal{A} $ serve as spatial growth sites. These atoms guide the placement of new scaffold atoms around them, ensuring both geometric alignment and chemical bonding constraints are satisfied. The anchors provide structural cues that reasonably guide the valid chemical expansion of the scaffold.

To model the molecular expansion process, we use a learnable function $ \phi $, which represents the diffusion dynamics. This function is implemented as a modified E(3)-equivariant Graph Neural Network (EGNN) [36]. At each time step $ t $, the input to the network consists of the noisy scaffold $\bar{F}_{\mathrm{t}}$ and the fixed context $ G^{C}_{S} $. These components are represented as a fully connected graph, where each node is characterized by its coordinate $ r $ and feature vector $ x $.

The model then predicts the noise $ \hat{\varepsilon } $, which consists of the coordinate and feature components. In order to make sure that the function $ \phi $ is invariant to translations, the initial coordinates ($\bar{F}_{t}^{r} $) are subtracted from the coordinate component of the predicted noise following Hoogeboom *et al.* [35]. Specifically, the predicted noise is given by:


(5) \begin{align*}& \hat{\varepsilon} = [\hat{\varepsilon}_{r}, \hat{\varepsilon}_{h}] = \phi(\bar{F}_{\mathrm{t}}, G^{C}_{S}, t) = \mathrm{EGNN}(\bar{F}_{t}, G^{C}_{S}, t) - [\bar{F}_{t}^{r}, 0].\end{align*}

The EGNN mainly consists of a sequence of E(3)-equivariant Graph Convolution Layers (EGCL), which is defined as:


(6) \begin{align*} m_{ij} &= \phi_{e}\bigl(x_{i}^{l}, x_{j}^{l}, d_{ij}^{2}, e_{ij}\bigr), \end{align*}

(7) \begin{align*} x_{i}^{l+1} &= \phi_{h}\bigl(x_{i}^{l}, \sum_{j \ne i} m_{ij}\bigr), \end{align*}

(8) \begin{align*} r_{i}^{l+1} &= \phi_{vel}(r_{i}^{l}, x_{i}^{l}, i), \end{align*}

(9) \begin{align*} \phi_{vel}(r_{i}^{l}, x_{i}^{l}, i) &= \begin{cases} \sum_{j \ne i}\frac{r_{i}^{l} - r_{j}^{l}}{d_{ij} + 1}\phi_{r}\bigl(x_{i}^{l}, x_{j}^{l}, d_{ij}^{2}, e_{ij}\bigr), & \mathrm{if}\ i \in \bar{F}_{t}, \\ 0, & \mathrm{if}\ i \in G^{C}_{S}. \end{cases} \end{align*}

where $ d_{ij} = \|r_{i}^{l} - r_{j}^{l}\| $. While a scalar distance encodes basic geometric information, it carries limited representational capacity and tends to be smoothed out by deep linear transformations during message passing. To enhance the model’s sensitivity to spatial details, we apply radial basis function (RBF) expansion to the interatomic distance $ d_{ij} $ by using a set of Gaussian basis functions:


(10) \begin{align*}& e_{ij}^{(k)} = \exp\left[-\frac{(d_{ij} - \mu_{k})^{2}}{2\sigma^{2}}\right], \quad k = 1, \dots, K.\end{align*}

Here: $\{\mu _{k}\}_{k=1}^{K}$ are fixed or learnable Gaussian centers (typically uniformly distributed over the interval $[0, d_{\max }]$); $\sigma $ is the shared bandwidth controlling kernel spread; the RBF feature vector $\mathbf{e}_{ij} = [e_{ij}^{(1)}, \dots , e_{ij}^{(K)}]^\top $ serves as a high-dimensional edge representation in the GNN. Each element $ e_{ij}^{(k)} $ is a value computed by the RBF, representing the response of the distance $ d_{ij} $ between node $ i $ and node $ j $ at the $ k $th Gaussian basis function.

Under this framework, the RBF expansion acts as a differentiable binning over the distance domain, allowing the network to explicitly distinguish interaction frequency bands across short-range and long-range neighbors. This helps preserve fine-grained geometric variations and long-range non-covalent interactions in protein–ligand binding pockets.

The functions $\phi _{e}$, $\phi _{h}$, and $\phi _{r}$ are all parameterized by neural networks.

**Message update**  $\phi _{e}$: The input is the concatenation of node embeddings $x_{i}^{l}$ and $x_{j}^{l}$, the squared distance $d_{ij}^{2}$ and the RBF $ e_{ij} $. Two fully connected layers with SiLU activation are conducted on the input to derive the output denoted by $m_{ij} \in \mathbb{R}^{n_{f}}$. The formal description is defined as follows.


(11) \begin{align*}& \mathrm{concat}[x_{i}^{l}, x_{j}^{l}, d_{ij}^{2},e_{ij}] \to \left\{ \begin{aligned} &\text{Fully connected layers} \\ &\to \mathrm{SiLU} \\ &\to \text{Fully connected layers} \\ &\to \mathrm{SiLU} \end{aligned} \right\} \to m_{ij}\end{align*}

**Feature update**  $\phi _{h}$: The input is the concatenation of node embedding $x_{i}^{l}$ and its aggregated message $m_{i} = \sum _{j} m_{ij}$. After the transformation defined as in Equation (12), the output is obtained as the updated node embedding.


(12) \begin{align*}& \mathrm{concat}[x_{i}^{l}, m_{i}] \to \left\{ \begin{aligned} &\text{Fully connected layers} \\ &\to \mathrm{BatchNorm} \\ &\to \mathrm{SiLU} \\ &\to \text{Fully connected layers} \\ &\to \mathrm{BatchNorm} \\ &\to \mathrm{add}(x_{i}^{l}) \end{aligned} \right\} \to x_{i}^{l+1}\end{align*}

**Coordinate update**  $\phi _{r}$: The input is the same as $\phi _{e}$, and the output is a scalar value defined as follows.


(13) \begin{align*}& \mathrm{concat}[x_{i}^{l}, x_{j}^{l}, d_{ij}^{2}, e_{ij}] \to \left\{ \begin{aligned} &\text{Fully connected layers} \\ &\to \mathrm{SiLU} \\ &\to \text{Fully connected layers} \\ &\to \mathrm{SiLU} \\ &\to \text{Fully connected layers} \end{aligned} \right\} \to \mathrm{output}\end{align*}

The equivariance of the convolutional layers with respect to the Euclidean group $E(3)$ is strictly preserved by design. Specifically, both the message passing function and the feature update function rely solely on scalar features and pairwise Euclidean distances, which are invariant under $E(3)$ transformations (including rotation and translation). Meanwhile, the coordinate update function is linear with respect to the relative positions of nodes, ensuring that the overall architecture remains equivariant under spatial transformations. In addition, the RBF distance encoding is also invariant under $E(3)$ transformations. Let the coordinates of two nodes $i$ and $j$ be $\mathbf{r}_{i}$ and $\mathbf{r}_{j}$, respectively. Under an $E(3)$ transformation consisting of a rotation $R \in \mathrm{SO}(3)$ and a translation vector $\mathbf{t} \in \mathbb{R}^{3}$, the coordinate transformation is defined as:


(14) \begin{align*}& \mathbf{r}_{i}^{\prime} = R\mathbf{r}_{i} + \mathbf{t}, \qquad \mathbf{r}_{j}^{\prime} = R\mathbf{r}_{j} + \mathbf{t}.\end{align*}

The pairwise Euclidean distance is preserved:


(15) \begin{align*}& d_{ij}^{\prime} = \|\mathbf{r}_{i}^{\prime} - \mathbf{r}_{j}^{\prime}\|_{2} = \|R(\mathbf{r}_{i} - \mathbf{r}_{j})\|_{2} = \|\mathbf{r}_{i} - \mathbf{r}_{j}\|_{2} = d_{ij},\end{align*}

since rotation preserves the norm. The Gaussian RBF also remains unchanged under transformation:


(16) \begin{align*}& e_{ij}^{(k)\,\prime} = \exp\left[-\frac{(d_{ij}^{\prime} - \mu_{k})^{2}}{2\sigma^{2}}\right] = e_{ij}^{(k)}.\end{align*}

Therefore, RBF features, used as scalar inputs in message and feature update functions, do not break the $E(3)$-equivariant structure of the network. Instead, they enhance its ability to encode fine-grained geometric information while strictly preserving equivariance.

After applying multiple EGCL layers, the updated graph consists of new coordinates $\hat{r} = [\hat{G}^{r} , \hat{\bar{F}}_{t}^{r}]$ and new features $\hat{x} = [\hat{G}^{x}, \hat{\bar{F}}_{t}^{x}]$. Since we are only interested in the predicted noise, we only take the tuple $[\hat{\bar{F}}_{t}^{r},\hat{\bar{F}}_{t}^{x}]$ as the final output of the EGNN [17].

### R-group generation stage

In contrast to the scaffold which serves as the structural skeleton of a molecule and defines its global geometry and core chemical features, R-groups primarily play the role in functional modification, spatial complementarity, and enhancing target specificity. These structures typically exhibit higher local complexity and structural diversity, significantly influencing molecular properties such as hydrophobicity, polarity, charge distribution, and geometric compatibility with the protein binding pocket. Therefore, incorporating R-groups is not merely a spatial extension of the scaffold but a critical step toward optimizing the molecule’s functional and pharmacological properties.

The R-group generation stage emphasizes the fine-grained modeling of local structural extensions, which is characterized by the following key aspects. First, R-group generation is conditioned on the prederived scaffold, where new atoms are added at positions determined by the model, based on spatial and chemical considerations. Second, to ensure spatial plausibility, the generated R-groups must align properly with the scaffold, avoid steric clashes, and maintain chemically reasonable growth directions. Finally, although explicit bond types and molecular graph topology are not modeled, the use of spatial guidance and geometric constraints allows the model to implicitly learn and preserve chemical validity at the coordinate level. To systematically achieve the generation and integration of R-groups, we further decompose this stage into the following steps.

#### Forward noise process of R-groups

In the forward noise injection process, noise is added to the atoms of the R-group. Inspired by previous methods [20], a virtual atom padding mechanism is introduced to support variable-length structure generation. Since most R-groups contain fewer than 10 atoms, each ligand is padded to a maximum of 10 atoms.

#### Reverse denoising of R-groups

During the reverse denoising step, the protein pocket and scaffold provide structural guidance for generating chemically valid R-groups. An E(3)-equivariant GNN predicts the atom coordinates and types. A polynomial noise schedule ensures stable generation, aligning the R-group with the scaffold and protein pocket constraints.

### Bond construction

In order to construct the chemical bonds between atoms, we utilize YuelBond [40], a multimodal framework based on GNNs, to accurately predict bond orders within molecules. Traditional methods, such as OpenBabel [41], typically rely on geometric and valence rules to infer molecular bond structures from atomic coordinates and features. However, these approaches often face limitations in practical applications, particularly when molecular geometries are distorted or imprecise, making it challenging to accurately reconstruct chemical bonds. As a result, predictions for molecular properties, such as SA and QED, tend to be less accurate, which are critical for drug design and other applications.

In this study, YuelBond is employed to effectively address these challenges by employing a graph-based learning approach that takes into account not only atomic connectivity but also local chemical environments and interatomic distances. Specifically, a foundational molecular graph is constructed by treating atoms as nodes and encoding their elemental types as node features. If the distance between two atoms is $\leq 3 $ Å, which is within the typical range for covalent bonds, an edge is formed between them, representing a potential chemical bond. The interatomic distance is then embedded as an initial edge feature. Next, an edge-focused iterative refinement process is employed to fine-tune these bond candidates, progressively refining them into meaningful bond representations. Each iteration involves three key steps: first, contextual information is constructed by combining the features of each atom with those of adjacent edges; second, edge features are updated by incorporating interatomic distances, capturing bond-type patterns while tolerating geometric distortions in noisy structures;finally, atomic node features are updated by aggregating neighboring information, ensuring each optimization step leverages the refined local environment. After these steps, the optimized edge representations are projected onto four main bond types (single bond, double bond, aromatic bond, andtriple bond) using a linear layer, and a softmax function is applied to generate the probability distribution for each category [40]. This enables YuelBond to make robust predictions even in the presence of noise or distortions in molecular geometries, providing more accurate molecular bonding representations, and improving the overall molecular quality accordingly.

### Decoupled training strategy

Instead of sharing parameters across both stages, we independently train the scaffold and R-group generators. Although parameter sharing is common in diffusion-based molecular generation, our experiments demonstrate that the parameter sharing causes the scaffold loss to dominate, hindering convergence, and degrading the R-group generation quality.

Thereafter, we adopt a decoupled training strategy where the two networks are trained separately with their own parameters and training data. Each model independently optimizes its reverse diffusion process, enabling balanced learning and stable convergence. We further apply gradient clipping to enhance numerical stability and prevent exploding gradients. The generation objective is to minimize the L2 loss between the true noise and the predicted noise:


(17) \begin{align*}& \mathcal{L} = \mathbb{E}_{t, G}\left[\|\epsilon - \hat{\epsilon}_\theta\|^{2}\right].\end{align*}

## Experiments

In this section, we first introduce the dataset and preprocessing procedure (including fragment acquisition and the cutting process of scaffold-R-group pairs), followed by a detailed description of experimental setup and result analysis.

### Dataset and preprocessing

#### Dataset collection

In experiments, we collected samples from the CrossDocked2020 dataset [42], which comprises $\sim $22.5 million protein–ligand complexes. For each complex, the ligand is associated with multiple receptor pockets curated from the Protein Data Bank (PDB), and docking is performed using the smina tool through the Pocketome pipeline. For each pocket, its corresponding ligands are docked against all candidate receptors. In order to ensure evaluation consistency with prior work [43], we collected samples with binding poses exhibiting a root-mean-square deviation (RMSD) <1 Å, reflecting high-quality docking conformations. To reduce redundancy and enhance data diversity, we further applied MMseqs2-based [44] clustering using a 30% sequence identity threshold. The final dataset includes 100 000 protein–ligand pairs for training and additional 100 samples reserved for testing.

#### Data preprocessing

**Figure 5:**
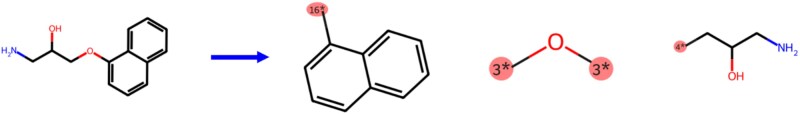
Illustration of BRICS-based molecular fragmentation.

In order to effectively train our framework, we use a BRICS-based molecular fragmentation strategy [45] (as shown in Fig. 5) to decompose each compound into a set of smaller structural fragments as starting points for molecule generation. Furthermore, to ensure the subsequent fragments can efficiently and controllably integrate with the generated portion, forming a complete scaffold, we select the molecular cleavage sites as growth anchors. At the same time, to ensure the chemical plausibility and structural applicability of these fragments, we further apply the “rule of three” principle [11–13] to filter the fragments, ensuring that each initial fragment possesses desirable drug-like properties and is suitable for structure-driven molecular design. Specifically, each fragment meets the following conditions.

- The molecular weight is <300 Da;
- The lipophilicity, measured by either logP (octanol–water partition coefficient) or logD at pH 7.4, is $\le $3;
- The number of hydrogen bond donors (e.g. N–H, O–H) and acceptors (e.g. N, O atoms) is no more than 3; the topological polar surface area (TPSA) is $\leq $ 60 Å$^{2}$; and the fragment contains no rotatable bonds.

**Figure 6:**
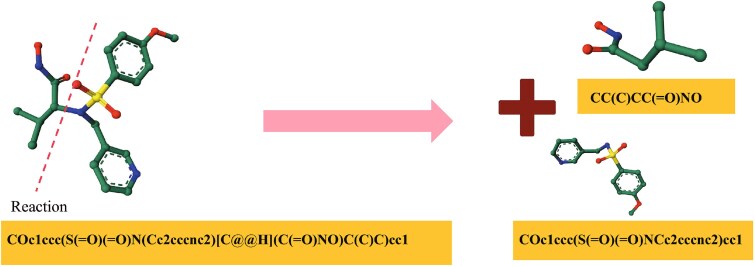
Illustration of the reaction-based molecular decomposition strategy used in LibINVENT.

Furthermore, to guarantee the diversity and synthesizability of generated molecules, we used LibINVENT [46] to slice each molecule into the pair of molecular scaffold and R-group. Specifically, LibINVENT is conducted based on 37 experimentally verified reaction SMIRKS templates (as shown in Fig. 6), covering a wide range of typical organic transformations and ensuring that each bond breakage corresponds to a chemically feasible reaction pathway. By applying LibINVENT, we obtain a training set with 76 108 tuples of molecular scaffolds, pockets, and R-groups, and a testing set with 43 tuples of molecular scaffolds, pockets, and R-groups, respectively.

We then applied the BRICS rule to further slice the molecular scaffolds to filter out chemically unreasonable molecules, ensuring the resulting fragments with better chemical plausibility and synthetic feasibility.

After the whole preprocessing procedure, we obtained a training set, a validation set, and a testing set with 50 403 samples, 12 601 samples, and 20 samples, respectively.

### Experimental setup

#### Hyper-parameter setting

In experiments, the proposed framework was implemented with 6 layers of EGNN, a hidden dimension of 128, a learning rate of 0.0002, a batch size of 32, and a total of 2000 training iterations.

#### Baselines

For performance comparison, we select the following three representative models as baselines.

- **Pocket2Mol** is an E(3)-equivariant generative network, which progressively predicts and adds new atoms based on the existing environment to derive a complete molecule.
- **FLAG** is a fragment-based ligand generation framework that generates 3D molecules segment by segment, and further refines the molecular geometry based on predicted rotational angles and structural optimization.
- **PMDM** is a conditional generative model that combines dual equivariant GNNs, capable of simultaneously capturing both local and global molecular dynamics, thereby efficiently generating drug-like molecules with reasonable binding affinity.

For a fair comparison, we use the same 20 protein pockets for the three baselines and our method for molecular generation. Due to the differences in methodology, Pocket2Mol and PMDM generate molecules from the atom-level, without involving the procedure of fragment processing. As FLAG, the core idea is based on fragment-based stepwise assembly. Although it also utilizes fragments, the generated molecules are assembled from a vocabulary built from the entire training set. In contrast, our method FDC-Diff is based on diffusion model, which focuses more on the growth process of initial fragments, which meet chemical plausibility and structural practicality.

#### Evaluation metrics

To systematically evaluate the properties and quality of generated molecules, we adopt several evaluation metrics which were widely used in prior studies [47]. These metrics cover diverse aspects, including binding performance, drug-likeness, and structural diversity.

- **Docking Affinity (Vina Score)** estimates the binding strength between the generated molecule and the target protein pocket using QVina, based on predicted molecular conformations and pocket interactions.
- **High Affinity** measures the proportion of generated molecules that exhibit higher binding affinity to the protein pockets compared with the reference ligands in the testing set.
- **Drug-likeness Score (QED)** evaluates the likelihood that a generated molecule serves as a drug candidate, with higher scores indicating stronger drug-likeness.
- **SA** reflects the ease of chemical synthesis, with normalized scores in the range [0,1]; higher values indicate easier synthesis.
- **Lipophilicity (LogP)** [48] represents the octanol–water partition coefficient of a molecule; the recommended range for drug-like compounds lies between −0.4 and 5.6.
- **Lipinski Rule Compliance (Lip.)** [49] counts how many of Lipinski’s “Rule of Five” criteria are satisfied, commonly used to assess oral drug-likeness.
- **Similarity to Training Set (Sim.)** computes the Tanimoto similarity between each generated molecule and its most similar counterpart in the training set, indicating the novelty relative to known compounds.
- **Structural Diversity (Div.)** quantifies the variation among generated molecules for each protein pocket, defined as 1 minus the average pairwise Tanimoto similarity, with higher values implying broader chemical exploration.

### Results and analysis

In this subsection, we comprehensively evaluate the performance of our proposed method and multiple baseline methods in the molecular generation task. Specifically, we analyze from two perspectives: the average performance of common generation indicators, and the structural characteristics of the generated molecules. Furthermore, we conduct ablation studies to investigate the contribution of main components in our proposed framework. The experimental results and analysis will be elaborated in detail as follows.

#### Evaluation on general metrics

For each target protein in the test set (consisting of a total of 20 target proteins), we generate 100 molecules for performance evaluation (with a total of 2000 molecules). Note that the sizes of generated molecules are sampled from the size distribution of the training set. The overall results of FDC-Diff and the baseline models are shown in Table 1. The results in Table 1 demonstrate that our model provides better performance on almost all evaluation metrics (except for similarity with the training set), compared to the baseline methods.

**Table 1:** Comparing the molecular properties of the test set and the generated molecules by different methods

Methods	Vina Score	High Affi.	QED	SA	LogP	Lip.	Sim.	Div.
	($\downarrow $)	($\uparrow $)	($\uparrow $)	($\uparrow $)		($\uparrow $)	($\downarrow $)	($\uparrow $)
Testset	−8.222 $\pm $ 0.38	−	0.492 $\pm $0.23	0.736 $\pm $0.15	3.012 $\pm $2.16	3.012 $\pm $2.16	−	−
PMDM	−8.062 $\pm $2.96	0.421 $\pm $0.24	0.576 $\pm $0.16	0.627 $\pm $0.15	2.212 $\pm $2.14	4.770 $\pm $0.47	**0.222** $\pm $0.01	0.895 $\pm $0.01
FLAG	−7.500 $\pm $2.50	0.413 $\pm $0.31	0.467 $\pm $0.19	0.672 $\pm $0.15	2.119 $\pm $2.21	4.510 $\pm $0.82	0.250 $\pm $0.07	0.896 $\pm $0.01
Pocket2 Mol	−8.554 $\pm $3.00	0.401 $\pm $0.38	0.558 $\pm $0.18	0.710 $\pm $0.14	2.221 $\pm $2.32	4.693 $\pm $0.60	0.248 $\pm $0.06	0.891 $\pm $0.01
FDC-Diff	**−8.730** $\pm $1.92	**0.563** $\pm $0.34	**0.643** $\pm $0.17	**0.729** $\pm $0.11	3.408 $\pm $1.64	**4.772** $\pm $0.49	0.286 $\pm $0.07	**0.897** $\pm $0.01

The best results are highlighted in bold. Upward arrows indicate that higher values are better, and downward arrows signify that smaller values are better.

To rigorously validate the performance gains of FDC-Diff, we performed a statistical hypothesis test (In our study, the Wilcoxon signed-rank test [50, 51] was utilized) across the 20 test pockets against three baseline models, and the results are presented in Table 2. The results in Table 2 clearly show that the performance improvement of FDC-Diff over three baseline models is indeed significant across all three metrics. Therefore, these observations confirm that FDC-Diff not only surpasses the current state-of-the-art models in key pharmacological properties but also maintains robust performance across various evaluation metrics.

**Table 2:** Results of statistical test on SA, QED, and Vina scores

Comparison	SA		QED		Vina Score	
	$P$ -value	Significance	$P$ -value	Significance	$P$ -value	Significance
FDC-Diff vs PMDM	9.536e-07	✓	0.0133	✓	1.335e-05	✓
FDC-Diff vs FLAG	0.0001	✓	2.861e-06	✓	0.0036	✓
FDC-Diff vs Pocket2Mol	0.0332	✓	0.0003	✓	0.0045	✓

**Figure 7:**
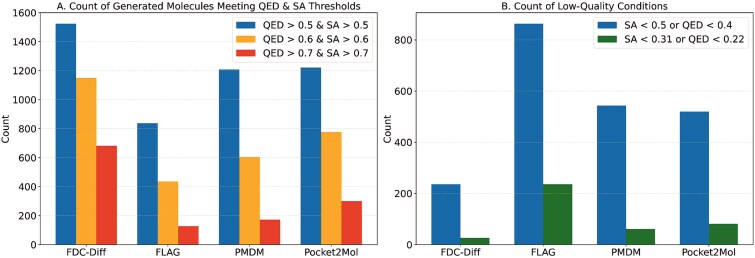
Number of molecules simultaneously satisfying different SA and QED thresholds.

It is worth noting that TAGMol [52] sets property thresholds based on the property distribution observed in currently marketed drugs. According to its model description and the results of Ziv *et al.* [53], molecules with specific properties below a certain threshold are generally not considered “high-value” candidate molecules. In our study, we observed that molecules generated by other methods often exhibit highly unbalanced scores (e.g. as shown in Fig. 7), some molecules exhibit extremely high SA scores, but low QED scores, and vice versa), and many generated molecules are meaningless or unreasonable. In contrast, due to incorporating prior knowledge in the process of molecule generation, the proposed method FDC-Diff is more effective in maintaining structural rationality.

#### Quantitative comparison of generated and reference molecules

**Figure 8:**
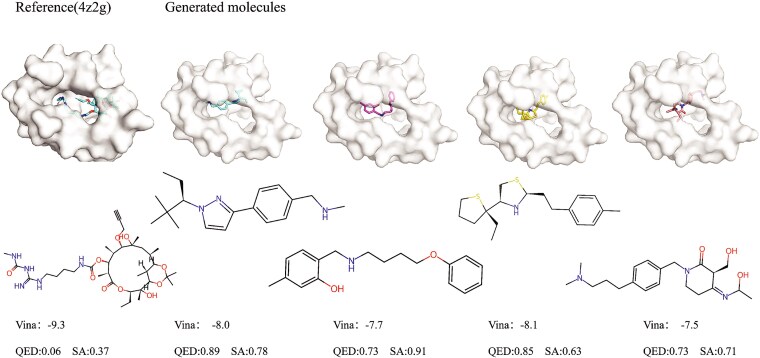
Docking performance comparison between the reference molecule and generated molecules on the protein $4z2g$.

**Figure 9:**
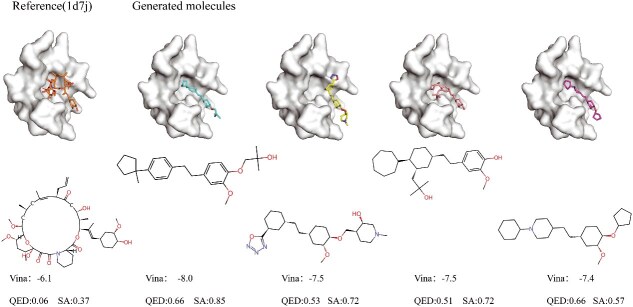
Docking performance comparison between the reference molecule and generated molecules on the protein $1d7j$.

In this subsection, we selected two target proteins (i.e. 4z2g and 1d7j) as examples to quantitatively evaluate the generated molecules, and the results are shown in Fig. 8 and Fig. 9, respectively. For each target protein, we selected four generated molecules by our model, and we compare the docking performance of the generated molecules with the reference molecules.

We find that the reference molecules are usually macrocyclic compounds with low conformational strain, which can bind well to the protein pockets, but they have very limited practical value due to poor SA and minimal drug-likeness. The generated molecules perform better than reference molecules according to the overall performance, which exhibit significant advantages in both SA and QED. This observation further validates that our model can generate novel molecules to bind with target proteins, rather than merely modifying the reference molecules.

When evaluating the overall quality of generated molecules, conventional metrics provide a certain degree of reference, but a deeper understanding of model performance requires attention to local structural features. To this end, we conducted the following experiments and analyses.

#### Atomic composition analysis

**Figure 10:**
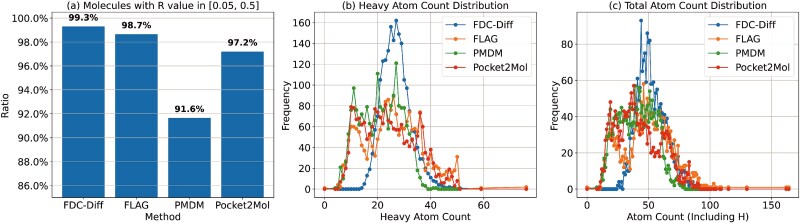
Distributions of (non-CH/non-H) ratios, heavy atom count, and total atom count for molecules generated by our model and baselines.

Previous studies [54, 55] suggest that the $R\ value$ of a candidate drug (which is defined as the ratio of non-carbon–hydrogen atoms to all non-hydrogen atoms) should generally fall within the range [0.05–0.50]. As shown in Fig. 10a, our method achieved superior performance according to this metric, for which $\sim $99.3% generated molecules fall within the desirable range.

In addition, the Retro Drug Design framework [56] emphasizes that most approved CNS small-molecule drugs typically contain $\sim $27–37 heavy atoms. Moreover, several studies [48, 57] have proposed that the total number of atoms (including hydrogen) in drug-like compounds should generally fall within the range of 20–70. Our model also adheres well to these structural criteria, which generates a substantial number of molecules that fall within these optimal ranges (shown in Fig. 10b and c).

#### Ring structure analysis

**Figure 11:**
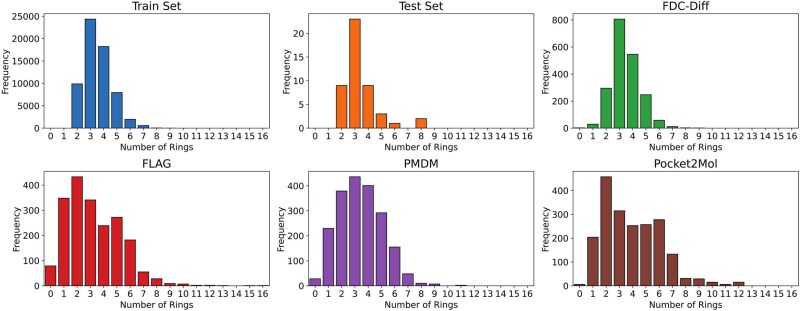
Distributions of the number of rings in generated molecules by our model and baselines.

As shown in Fig. 11, we also analyzed the distribution of the number of rings in generated molecules across all methods, as well as in the training and testing sets. The distribution produced by our model aligns closely with that of the training and test sets, indicating that it effectively captures the true data distribution from a local structural perspective.

#### Statistical divergence of bond length and torsional angle distributions

**Figure 12:**
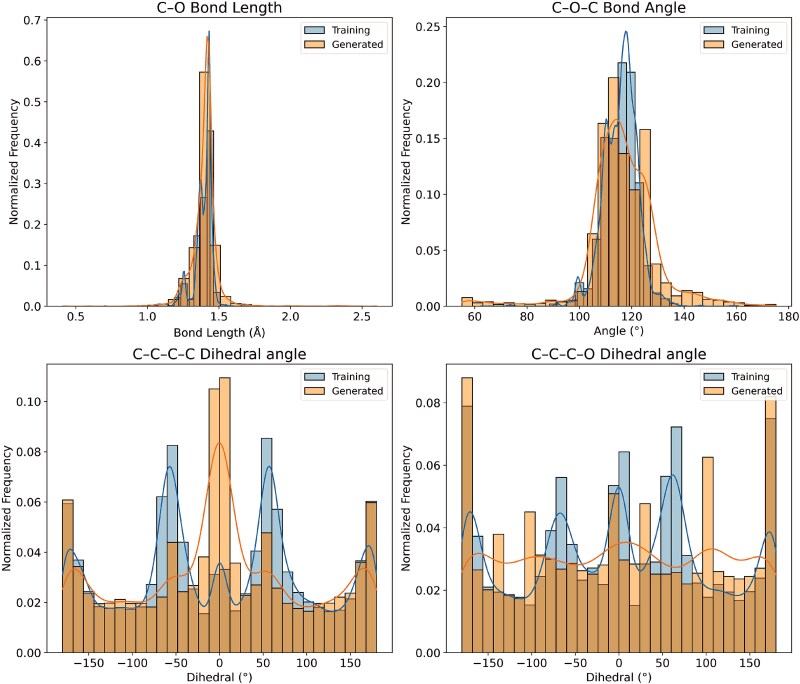
Distributions of C–O bond lengths, C–O–C bond angles, C–C–C–C dihedral angles, and C–C–C–O dihedral angles in generated molecules and training samples.

**Figure 13:**
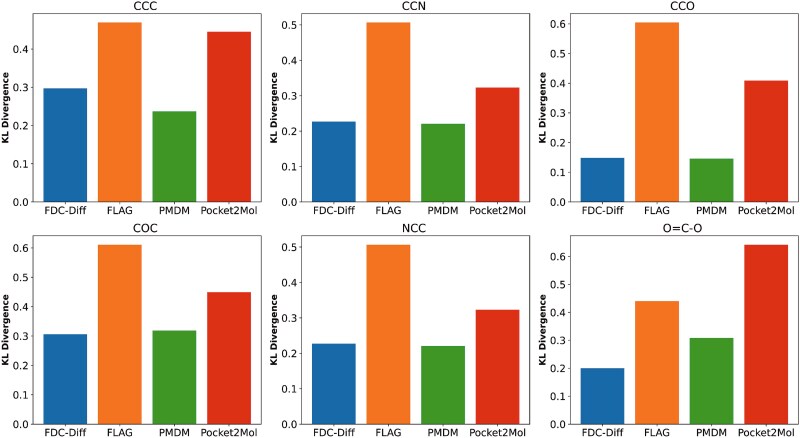
Comparison between our model and baselines on KL divergence of common bond angles.

**Figure 14:**
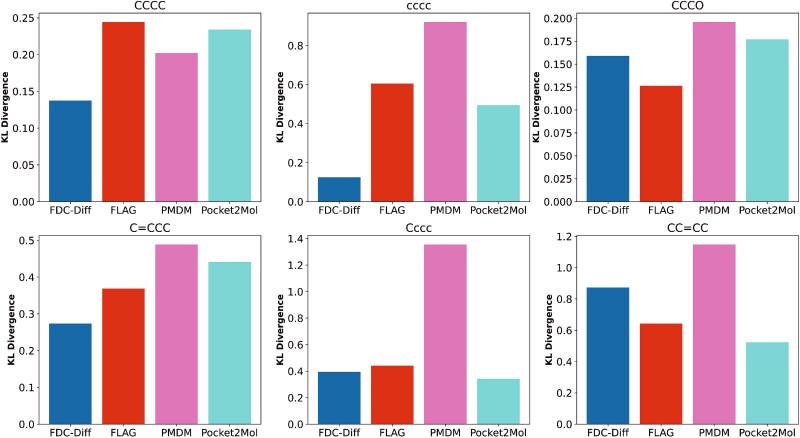
Comparison between our model and baselines on KL divergence of common dihedral angles.

In addition, we analyzed the distributions of bond lengths, bond angles, and dihedral angles in the generated molecules, comparing with those in the training set (as shown in Fig. 12). Note that we use the RDKit to calculate bond angles and dihedral angles in radians. Furthermore, we measured the KL divergence between the distributions of common bond angles and dihedral angles in molecules generated by different methods and those in the training set (as shown in Figs 13 and 14). A lower KL divergence indicates better model performance.

The results demonstrate that, in terms of bond angle distributions (shown in Fig. 13), our method shows lower divergence than baseline methods for most angle types (except for $CCC$). Regarding dihedral angle distributions, moreover, our method outperforms the baseline methods for most types (shown in Fig. 14). These observations suggest that our model is able to effectively preserve geometric features, generating structurally reasonable molecules accordingly.

In summary, our method not only achieves balanced performance across multiple drug-likeness metrics but also demonstrates clear advantages in terms of structural rationality, showcasing stronger generalizability and high likelihood of producing potential drug candidates.

#### Ablation study

**Figure 15:**
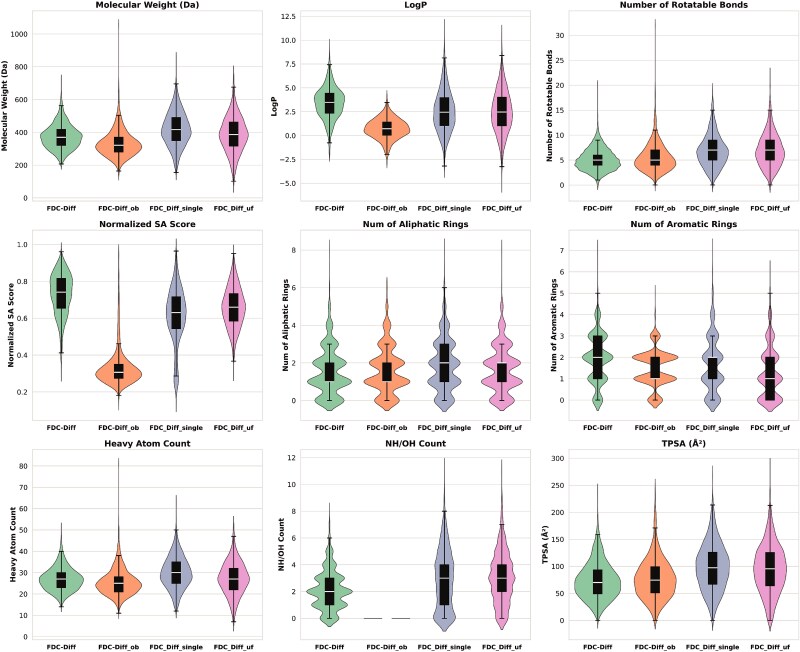
Chemical property distributions of molecules generated by FDC-Diff and three variants in the ablation study.

In this section, we conduct an ablation study to investigate the contributions of three main components in our model (i.e. Rule-of-Three (Ro3) fragment constraint, the adaptive assembly mechanism, and the decoupled training strategy) to the performance improvement on the task of fragment-based molecular generation. More specifically, three variants of FDC-Diff are built for performance comparison. “FDC-Diff_uf” denotes the variant without the filtering process, i.e. the Rule-of-Three (Ro3) fragment constraint of FDC-Diff is removed and replaced by random initial fragment selection; “FDC-Diff_ob” denotes the variant in which the YuelBond mechanism is replaced by the OpenBabel assembly; “FDC-Diff_single” denotes the variant in which the decoupled training strategy is replaced by a single-stage generation process. The comparison results of evaluation metrics are presented in Table 3, and the distributions of key physicochemical properties are visualized in Fig. 15.

**Table 3:** Results of ablation study

Methods	Vina Score	High Affi.	QED	SA	LogP	Lip.	Div.
	($\downarrow $)	($\uparrow $)	($\uparrow $)	($\uparrow $)		($\uparrow $)	($\uparrow $)
FDC-Diff_ob	−7.782 $\pm $ 1.80	0.269 $\pm $0.28	**0.680** $\pm $0.14	0.331 $\pm $0.10	0.708 $\pm $1.06	**4.908** $\pm $0.35	0.875 $\pm $0.01
FDC-Diif_uf	−7.593 $\pm $1.89	0.251 $\pm $0.28	0.459 $\pm $0.19	0.659 $\pm $0.11	2.526 $\pm $2.21	4.276 $\pm $0.87	0.887 $\pm $0.01
FDC-Diff_single	−7.980 $\pm $2.02	0.369 $\pm $0.30	0.455 $\pm $0.20	0.6134 $\pm $0.15	2.573 $\pm $2.16	4.373 $\pm $0.90	0.894 $\pm $0.02
FDC-Diff	**−8.730** $\pm $1.92	**0.563** $\pm $0.34	0.643 $\pm $0.17	**0.729** $\pm $0.11	3.408 $\pm $1.64	4.772 $\pm $0.49	**0.897** $\pm $0.01

Upward arrows indicate that higher values are better, and downward arrows signify that smaller values are better.

From Table 3 and Fig. 15, we can find that our model FDC-Diff performs better than all three variants by considering all evaluation metrics and all physicochemical properties, indicating the necessity of integrating these components for generating high-quality drug candidates.

First, “FDC-Diff_uf” generated molecules with a high average of rotatable bonds (with average 6.95 and median 7.0) and the fewest aromatic rings among the methods. Since its training and testing sets include fragments that do not comply with the Ro3 rule, this method may generate molecules with MW > 500 and TPSA >140 compared with our approach. These results indicate the importance of the Ro3 constraint for maintaining structural validity and reserving sufficient chemical space.

Second, “FDC-Diff_ob” exhibits a severe deterioration in structural validity and SA (with the average SA score 0.33) despite achieving a relatively higher QED. The generated molecules display chemically implausible features: a disproportionate bias toward aliphatic rings over aromatic systems, a complete absence of hydrogen bond donors (NH/OH count of 0), and an atypically low LogP (0.7083). This structural bias stems from a limitation in OpenBabel’s deterministic bond perception algorithms. In contrast, YuelBond employs an edge-focused iterative refinement. By aggregating contextual node and edge features rather than relying solely on raw geometry, YuelBond accurately infers bonds despite structural distortions, ensuring the generation of chemically valid structures.

Third, “FDC-Diff_single” generated molecules with higher molecular weights and TPSA, along with excessive rotatable bonds (average 6.83). Notably, this method yielded the highest number of aliphatic rings, resulting in structures with high conformational flexibility. The possible reason is that for molecular generation requiring precise topology control, the decoupled strategy is essential to balance global scaffold generation with local functional refinement, thereby avoiding the entropic penalty associated with single-stage methods.

In summary, the three main components in FDC-Diff are all essential for the effectiveness of the proposed framework, ensuring a superior balance between structural validity, SA, and pharmacological properties.

#### Lead compound generation

In this section, we apply the proposed model to the task of lead compound generation to evaluate its effectiveness for real-world drug development.

Alzheimer’s disease (AD) is a progressive neurodegenerative disorder that leads to cognitive decline, memory loss, and impaired daily functioning, affecting millions of people worldwide. Currently, acetylcholinesterase inhibitors (AChEIs) are the primary therapeutic option for managing cognitive deficits in mild to moderate AD. These inhibitors work by blocking acetylcholinesterase (AChE), which increases acetylcholine levels in the brain and enhances cholinergic neurotransmission, thereby improving cognitive function. Although their efficacy is established, current AChEIs face significant limitations. First, the therapeutic benefits are modest and fail to halt disease progression, offering only symptomatic relief that diminishes over time. Furthermore, these drugs are associated with various adverse effects, including gastrointestinal disturbances such as nausea and diarrhea, cardiovascular risks such as bradycardia and syncope, and neuropsychiatric symptoms such as anxiety, aggression, and hallucinations. These side effects often necessitate dose reductions and restrict tolerability.

**Figure 16:**
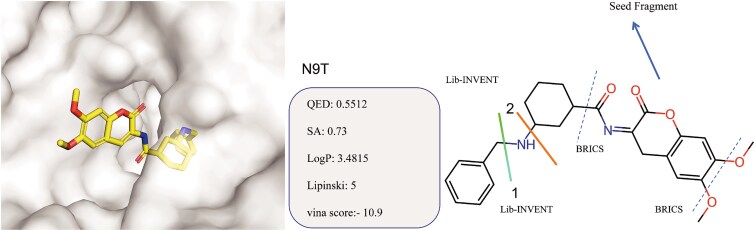
The ligand N9T in the AChE crystal structure of PDB 6TT0 and its physicochemical properties. Fragmentation strategy for N9T: First, reaction rules were applied to cleave the molecule at Sites 1 and 2, respectively, yielding two distinct sets of scaffold-R-group pairs. Subsequently, the BRICS rules were employed to further decompose these scaffolds, isolating an identical seed fragment as the start of generation process.

To address these challenges and further explore the practical significance of FDC-Diff, we applied the model to generate high-affinity molecules targeting the AChE enzyme. We utilized a subunit from the crystal structure of the AChE chiral inhibitor reported by Catto [58] and colleagues (PDB ID: 6TT0) and analyzed it with the corresponding inhibitor MC1420 (labeled as N9T in the crystal) as shown in Fig. 16. Although MC1420 serves as an efficient reversible dual-binding site AChE inhibitor with nanomolar affinity (IC50 = 19.2 nM) and good selectivity for butyrylcholinesterase at 730-fold, its X-ray crystal structure resolution of only 2.8 Åprevents clear determination of the absolute configuration and requires additional docking simulations for support. Furthermore, prolonged exposure in cytotoxicity tests reveals potential toxicity, which may limit its further application in drug development.

**Figure 17:**
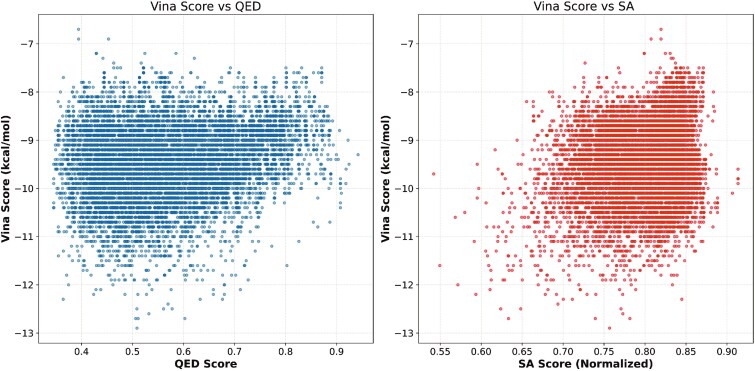
Illustration of three key properties of generated molecules.

In this study, we applied FDC-Diff to fragment the inhibitor and obtained seed fragments along with scaffold-R-group pairs. These seed fragments were assembled with denoised generated components to produce 10 000 optimized molecules. As illustrated in Fig. 17, we plotted the distribution of three key properties (i.e. QED, SA, and Vina scores) for the generated molecules. The results show that all molecules have good Vina scores and SA scores, while the QED scores are moderate. Overall, the proposed method FDC-Diff holds significant promise in drug discovery by effectively producing molecules with excellent drug-likeness, SA, and binding affinity, thereby offering new candidate compounds for treating Alzheimer’s disease.

**Figure 18:**
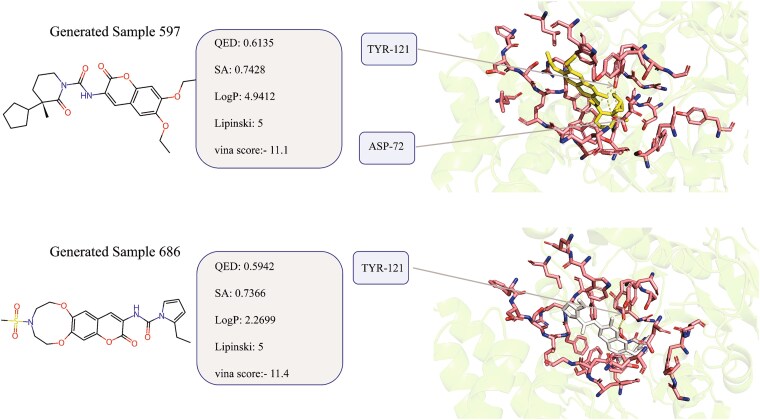
Illustration of two generated molecules with desirable properties.

In addition, to further investigate the quality of the generated molecules, we selected two compounds with desirable properties (as shown in Fig. 18). From Fig. 18, we found that it forms hydrogen bonds with the ASP-72 and TYR-121 amino acid residues in the target protein pocket. This type of molecular interaction typically enhances binding stability. In contrast, the original ligand (shown in Fig. 16) does not form direct hydrogen bonds with the protein. Hydrogen bonds, as a crucial type of molecular interaction, can improve the binding stability between the ligand and receptor, potentially leading to more durable binding in drug design. Therefore, these characteristics demonstrate the effectiveness of the proposed method for deriving feasible or potential candidates in real-world drug development.

## Conclusions and future work

In this paper, we have proposed a fragment-based dual conditional diffusion generation framework, FDC-Diff. The model starts with seed fragments to first progressively grow a reasonable molecular scaffold, and then completes the substituents step by step based on this scaffold, ultimately generating a realistic 3D molecule. Based on the comprehensive experimental results, FDC-Diff demonstrates superior overall performance on standard molecular generation benchmarks, and significantly outperforms existing baseline methods in generating structures with potential drug-like properties.

Although achieving the best performance, the proposed model still has two limitations. The first limitation lies in the restricted expressive power of its underlying GNN architecture, which may cap its ability to capture intricate molecular interactions. Second, our model relies on atomic diffusion coupled with a heuristic bond inference by applying the YuelBond. By inferring bond types based on static distances rather than explicitly modeling bond formation, the proposed model becomes sensitive to minor coordinate deviations, occasionally yielding chemically invalid structures.

In future work, first, we plan to develop a unified joint diffusion framework that simultaneously evolves atomic coordinates and bond topologies, thereby better capturing the true distributional properties of molecular graphs. Second, we will integrate rigorous physicochemical priors into protein–ligand modeling to ensure generated compounds possess structural and functional realism. Third, moving beyond traditional metrics like binding affinity or SA, we plan to adopt multi-modal evaluation strategies akin to the DFT-ANPD framework [59], which will allow us to assess both structural and semantic validity, steering molecule generation toward clinical drug standards. Finally, to mitigate the “black box” nature of current models, we will explore the integration of Kolmogorov–Arnold Networks to enhance the mechanistic interpretability, offering clearer insights into the decision-making process and facilitating trust in drug design applications.

**Key Points**

- A fragment-based dual conditional diffusion framework is proposed for 3D molecular structure generation. As far as we know, this is the first attempt to introduce diffusion-based generative model to the task of fragment-based drug design.
- The coordinated dual-diffusion architecture is able to effectively model distinctive semantics contained in scaffold and R group, respectively.
- Extensive experiments verify the effectiveness of the proposed method for the molecular generation task.

## Data Availability

The data and source codes are available in GitHub at https://github.com/CHT713/FDC-Diff.
